# Long-Term Inhibition of Xanthine Oxidase by Febuxostat Does Not Decrease Blood Pressure in Deoxycorticosterone Acetate (DOCA)-Salt Hypertensive Rats

**DOI:** 10.1371/journal.pone.0056046

**Published:** 2013-02-05

**Authors:** Theodora Szasz, Robert Patrick Davis, Hannah S. Garver, Robert J. Burnett, Gregory D. Fink, Stephanie W. Watts

**Affiliations:** 1 Department of Physiology, Georgia Health Sciences University, Augusta, Georgia, United States of America; 2 Department of Pharmacology and Toxicology, Michigan State University, East Lansing, Michigan, United States of America; Maastricht University, The Netherlands

## Abstract

Xanthine oxidase and its products, uric acid and ROS, have been implicated in the pathogenesis of cardiovascular disease, such as hypertension. We have previously reported that allopurinol inhibition of XO does not alter the progression of deoxycorticosterone acetate (DOCA)-salt hypertension in rats. However other researchers have observed a reduction in blood pressure after allopurinol treatment in the same model. To resolve this controversy, in this study we used the newer and more effective XO inhibitor febuxostat, and hypothesized that a more complete XO blockade might impair hypertension development and its end-organ consequences. We used DOCA-salt hypertensive rats and administered vehicle (salt water) or febuxostat (orally, 5 mg/kg/day in salt water) in a short-term “reversal” experiment (2 weeks of treatment 3 weeks after DOCA-salt beginning) and a long-term “prevention” experiment (treatment throughout 4 weeks of DOCA-salt). We confirmed XO inhibition by febuxostat by measuring circulating and tissue levels of XO metabolites. We found an overall increase in hypoxanthine (XO substrate) and decrease in uric acid (XO product) levels following febuxostat treatment. However, despite a trend for reduced blood pressure in the last week of long-term febuxostat treatment, no statistically significant difference in hemodynamic parameters was observed in either study. Additionally, no change was observed in relative heart and kidney weight. Aortic media/lumen ratio was minimally improved by long-term febuxostat treatment. Additionally, febuxostat incubation in vitro did not modify contraction of aorta or vena cava to norepinephrine, angiotensin II or endothelin-1. We conclude that XO inhibition is insufficient to attenuate hypertension in the rat DOCA-salt model, although beneficial vascular effects are possible.

## Introduction

Xanthine oxidase (XO) is a ubiquitous enzyme essential in the last steps of purine catabolism, the end-product of which, uric acid, has been independently associated with risk for cardiovascular and kidney disease [1], [2]. Besides uric acid, XO also generates reactive oxygen species (ROS), specifically superoxide/hydrogen peroxide which serve as important signaling molecules in cardiovascular tissues. Increased ROS levels are a well known pathogenetic factor in hypertension, be it experimentally induced in animals or essential/secondary hypertension in humans [3]. Excessive amounts of ROS in tissues also can cause injury, and studies aimed at understanding hypertension-related tissue damage have shown increases in XO expression or activity in animal models of hypertension such as DOCA-salt and the spontaneously hypertensive rat [4], [5], [6]. A study of genetic variations in the xanthine dehydrogenase gene in a population of male Japanese subjects [7] found significant associations of SNPs in this gene with hypertension, carotid atherosclerosis and chronic kidney disease, suggesting that mutations of this gene may contribute to hypertension and its complications. Beneficial effects of XO inhibition have been observed in cardiomyopathies, hypertension and associated target organ damage, as well as aging [8]. Conversely, uric acid has been shown to decrease NO bioavailability, thus potentially impairing endothelial function [9]. Furthermore, hyperuricemia is associated with endothelial dysfunction in humans [10] and experimentally increasing uric acid levels has lead to hypertension and renal damage in rats [11], [12].

However, despite the known association between hyperuricemia and hypertension, a clear cause-effect relationship could not yet be established. One approach to this issue has been to observe the effects of XO inhibition on blood pressure. The drug allopurinol has long been used clinically in the chronic treatment of gout and other hyperuricemia-associated disorders. Clinically, allopurinol was only shown to decrease blood pressure when administered to hyperuricemic patients [13]. Furthermore, studies using allopurinol in rodent models of hypertension have generated largely negative results, in that allopurinol did not reduce blood pressure. However conflicting results have been observed depending on the specific hypertension model and/or the dose of the drug used [14], [15], [16], [17], [18], [19]. Contradicting most of these results, one study in particular [15] has reported that allopurinol decreases systolic blood pressure in the rat DOCA-salt hypertensive model by an impressive average of ∼40 mmHg. However, using the same model and only slight methodology differences, we previously reported that allopurinol does not decrease blood pressure, nor prevent hypertension development in DOCA-salt hypertensive rats [20]. However our results could be theoretically questioned, based on a possibly insufficient degree of XO inhibition by allopurinol. Febuxostat is a new, selective and more efficacious inhibitor of XO activity that lacks a purine structure and acts via a different mechanism of action than allopurinol [21], [22]. Clinical studies have demonstrated the superior effects of febuxostat compared to allopurinol in gout patients [23], [24], [25], [26], in which febuxostat is administered in cases with renal or hepatic impairment [27]. Currently there are no reports on the effects of febuxostat in animal models of hypertension, other than hyperuricemia-induced hypertension, where lowering uric acid by febuxostat resulted in amelioration of hypertension and renal alterations [28], [29], [30]. In the present study, we evaluated the effects of febuxostat administration on both developing and established hypertension in the rat DOCA-salt hypertension model. Thus, our approach was to use a compound that is structurally distinct from allopurinol in order to resolve the issue of whether XO inhibition results in a fall in blood pressure in a model of hypertension that is not associated with hyperuricemia.

## Methods

### Animal Use

All animal procedures were approved by and performed in accordance with regulations of the Institutional Animal Care and Use Committee at Michigan State University. Male Sprague-Dawley rats (225–250 g, Charles River Laboratories, Indianapolis, IN) were used. Rats were fed standard rat chow (Teklad®) and given water *ad libitum* until the start of DOCA-salt treatment. Euthanasia was performed with pentobarbital (60–80 mg/kg i.p.).

### Radiotelemetric Measurement of Mean Arterial Pressure (MAP)

Under isoflurane anesthesia, radiotelemeter devices (TA11PA-C40, Data Sciences International, St. Paul, MN) with attached catheters with pressure-sensing tips were implanted into a subcutaneous pouch in the abdominal wall through a 1–1.5 cm incision in the left inguinal area. Catheters were introduced into the left femoral artery and the tip was advanced to the abdominal aorta. Rats were allowed at least 3 days to recover postoperatively. Systolic pressure, diastolic pressure, mean arterial pressure, pulse pressure, heart rate and activity (movement) were recorded throughout the duration of the study at a sampling rate of 10 seconds each 10 minute interval (Dataquest ART 4.1, DSI). Data are presented as mean ± SEM of 24 h-averaged parameters.

### DOCA-salt Hypertension

Rats were uninephrectomized and received a deoxycorticosterone acetate (DOCA) subcutaneous implant (150 mg/kg). For the rest of the duration of the study, rats were given drinking water supplemented with 1% NaCl and 0.2% KCl.

### Febuxostat Administration and Biological Sample Collection

Febuxostat (2-(3-cyano-4-isobutoxyphenyl)-4-methyl-1,3-thiazole-5-carboxylic acid) was obtained from Takeda Pharmaceuticals U.S.A., Inc. Two treatment protocols were performed, with N = 8 in each group. In both protocols, radiotelemeters were first implanted in rats and then a recovery period of at least 3 days was followed by a 3-day period of baseline recording. In the short-term study, DOCA surgery was performed following baseline recording. After 3 weeks of DOCA-salt treatment, rats were divided into control and febuxostat groups, receiving salt water only or salt water containing febuxostat, respectively, for 2 weeks. In the long-term study, rats were divided into control and febuxostat groups immediately following baseline recording and received tap water or tap water containing febuxostat, respectively, for 3 days. DOCA surgery was performed next, followed by 4 weeks of salt water administration for the control group or salt water containing febuxostat for the febuxostat group. In both protocols, febuxostat concentrations in the drinking water were adjusted bi-weekly according to monitored water intake and body weight in order to maintain a constant dosage of 5 mg/kg/day febuxostat. The 24 h water intake itself was not significantly different between control and febuxostat-treated rats in either study.

At the end of both studies, rats were moved to metabolic cages for 24 h urine collection. Rats were then sacrificed under anesthesia with pentobarbital (60–80 mg/kg i.p.), and blood and tissue samples (aorta, vena cava, carotid artery, jugular vein, liver, kidney, heart) were collected. Serum was separated from blood samples and tissues were cleaned in physiological salt solution (PSS) containing (mM): NaCl, 130; KCl, 4.7; KH_2_PO_4_, 1.18; MgSO_4_·7H2O, 1.17; NaHCO_3_, 14.8; dextrose, 5.5; CaNa_2_EDTA, 0.03; CaCl_2_, 1.6. A segment of aorta was saved for histological analysis. The heart and the right kidney were wet-weighed. Heart and kidney weight were reported as percentage of total body weight. Total body weight itself was not changed as a result of febuxostat treatment.

### High Performance Liquid Chromatography (HPLC)

Serum and urine samples were diluted 1∶10 in 0.2 M acetic acid. Tissues were quick frozen in liquid nitrogen, pulverized, solubilized in 0.2 M acetic acid and sonicated. All samples were then centrifuged (14,000 g for 10 min at 4°C) and supernatants were used for analysis of XO metabolites using a Waters HPLC (Waters, Milford, MA) coupled with Photo Diode Array detection. Separation of analytes was achieved on a Phenomenex Luna, 5 µm, C-18, 250×4.6 mm column with a 0.02 M sodium acetate mobile phase. A gradient with methanol was used to clear the column after elution of analytes. The individual XO metabolites were analyzed at their respective maximum wavelength intensities (uric acid at 284 nm, hypoxanthine at 249 nm). Febuxostat was measured with fluorescence detection (excitation 320 nm, emission 380 nm) on a Dionex MD-150 column with a mobile phase of 0.032 M acetic acid/acetonitrile (55∶45). Peak height values for each metabolite obtained in the same retention time window as standards were used as input into standard curves constructed with known analyte concentrations. Analytes were normalized to total protein content of HPLC samples determined by the Bradford method, with the exception of urine, for which they were normalized to the total urine volume for 24 h.

### Histology

Cross-sections of thoracic aorta segments collected at the time of sacrifice were paraffin-embedded and stained with hematoxylin and eosin. Morphometric assessment was performed using the MMI Cell Tools software (Molecular Machines and Industries, Glattbrugg, Switzerland) on a Nikon Eclipse microscope. Equally spaced (every 45°) measurements of lumen diameter (4 measurements) and wall thickness (8 measurements) were made. The averaged wall thickness was divided by the averaged lumen diameter to calculate a final wall/lumen ratio.

### Isolated Tissue Bath Contractility

Thoracic aortic and inferior vena cava rings were cleaned and mounted in warmed (37°C), aerated (95% O_2_, 5% CO_2_) PSS in isolated tissue baths for measurement of isometric contractile force. Cumulative concentration-response curves to norepinephrine, angiotensin II and endothelin-1 were constructed in the absence and presence of febuxostat (100 nm, 1 h). The initial contraction to agonist (10 μM phenylephrine for aorta, 10 μM norepinephrine for vena cava) was used to normalize any further response.

### Data Analysis

Data are presented as mean ± SEM for the number of animals (N). Plotting and statistical analysis of data was accomplished using GraphPad Prism 5 (GraphPad, La Jolla, CA). When comparing groups, the appropriate Student’s *t*-test or ANOVA analysis was performed. For *in vivo* experiments, a 2-way repeated measures ANOVA with Bonferroni *post-hoc* analysis was performed. In all cases, a *p* value of ≤0.05 was considered statistically significant.

## Results

### Febuxostat Treatment did not Significantly Change the Blood Pressure or Heart Rate of DOCA-salt Rats during Either Short or Long-term Administration

**Figure 1:**
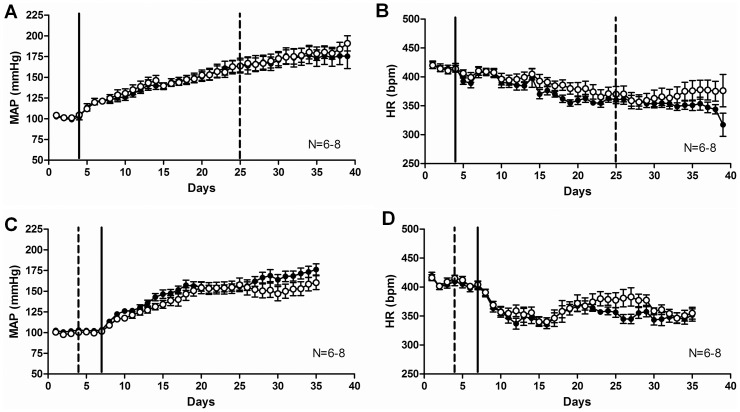
Febuxostat treatment did not significantly change the blood pressure or heart rate of DOCA-salt rats in either short (A, B) or long (C, D)-term administration. Radiotelemetric measurements of mean arterial pressure (A, C) and heart rate (B, D) of control (full symbols) and febuxostat-treated (open symbols) DOCA-salt rats. Data are expressed as means ± SEM for N = 6–8. The continuous line represents the start of DOCA-salt treatment and the interrupted line represents the beginning of febuxostat treatment (21 days after DOCA-salt start in A, B and 3 days before DOCA-salt start in C, D).

To test the effects of XO inhibition by febuxostat at an established hypertension stage, febuxostat was administered to DOCA-salt rats as a short-term treatment for 14 days after full development of mineralocorticoid hypertension was obtained (day 21 of DOCA-salt hypertension). Hemodynamic parameters were recorded continuously via radiotelemetry. No statistically significant difference was observed in MAP (Fig. 1A), heart rate (Fig. 1B), systolic or diastolic pressures (last treatment day: control SBP = 209±11, febuxostat SBP = 215±9; control DBP = 148±10, febuxostat = 157±8; mmHg) between the control and febuxostat-treated groups.

To test the effects of XO inhibition by febuxostat on the development of hypertension, febuxostat was administered to DOCA-salt rats throughout the entire duration of DOCA-salt treatment. Hemodynamic parameters were recorded continuously via radiotelemetry. Despite an obvious trend for reduced MAP in the febuxostat group in the last week of the study, no statistically significant difference was observed in MAP (Fig. 1C), heart rate (Fig. 1D), systolic or diastolic pressures (last treatment day: control SBP = 204±10, febuxostat SBP = 186±10; control DBP = 151±5, febuxostat = 137±7; mmHg) between the control and febuxostat-treated groups.

### Both Short and Long-term Febuxostat Treatment Lowered Uric Acid and Increased Hypoxanthine Circulating and Tissue Levels in DOCA-salt Rats

**Figure 2:**
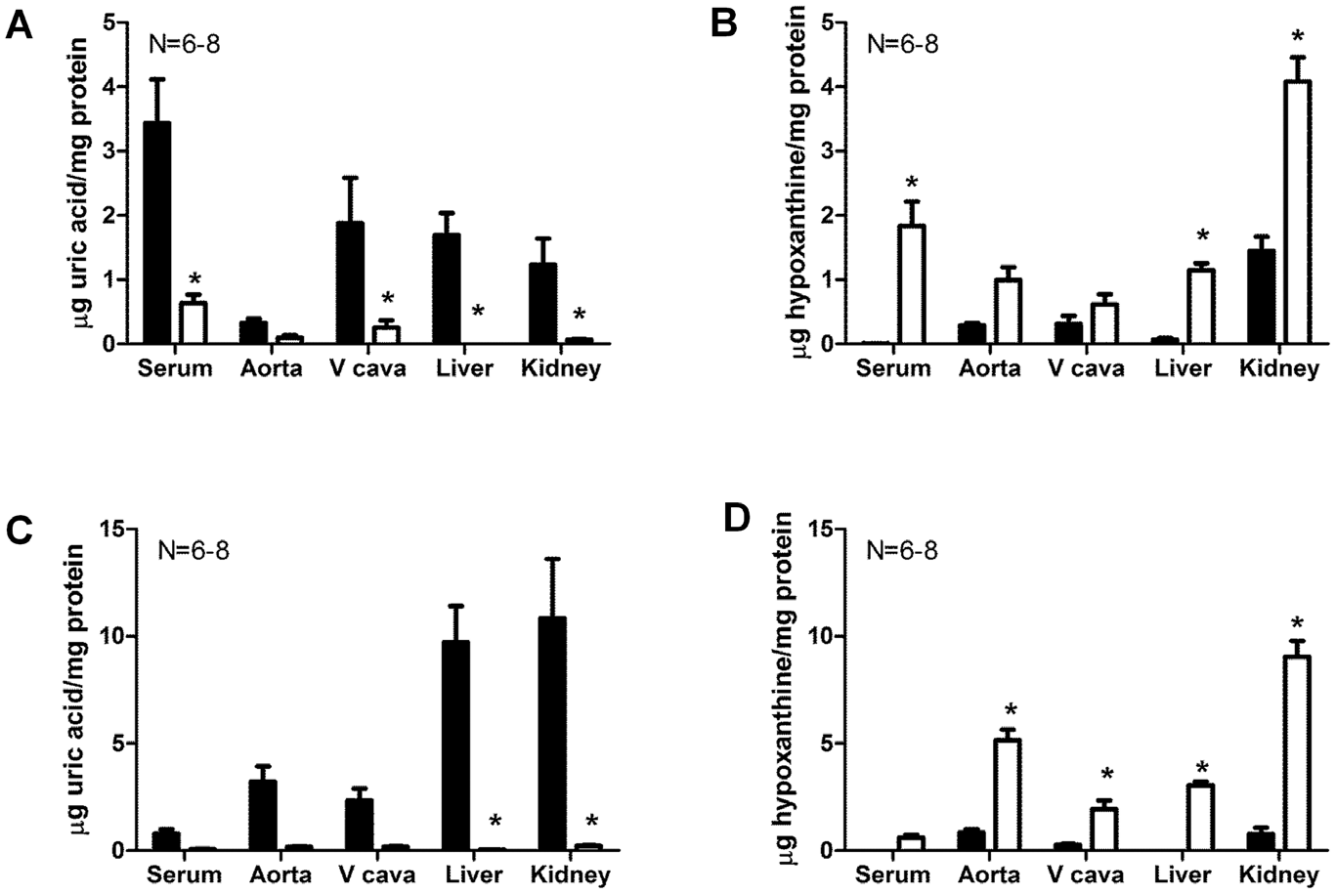
Both short and long-term febuxostat treatment lowered uric acid and increased hypoxanthine circulating and tissue levels in DOCA-salt rats. HPLC measurements of uric acid (A, C) and hypoxanthine (B, D) in DOCA-salt rats after short (A, B) or long (C, D)-term febuxostat administration. Data are expressed as means ± SEM for N = 6–8. *represents a statistically significant difference (p<0.05) between febuxostat and control rats.

Verification of xanthine oxidase inhibition by febuxostat was obtained by measurements of XO product uric acid as well as XO substrate hypoxanthine in the circulation and in tissues. These measurements revealed a general trend of reduction in uric acid levels and increase in hypoxanthine levels in all tissues (Table 1). Following short-term febuxostat administration, uric acid levels were significantly reduced in serum, liver, kidney and vena cava (Fig. 2A), while hypoxanthine levels were significantly increased in the serum, liver and kidney (Fig. 2B). Similarly, following long-term febuxostat administration, uric acid levels were significantly reduced in the liver and kidney (Fig. 2C), while hypoxanthine levels were significantly increased in the liver, kidney, aorta and vena cava (Fig. 2D). No significant difference was observed in urine levels of these metabolites (uric acid short-term: control = 1.281±0.199, febuxostat = 1.280±0.216; uric acid long-term: control = 1.504±0.166, febuxostat = 1.531±0.195; hypoxanthine short-term: control = 0.100±0.029, febuxostat = 0.335±0.048; hypoxanthine long-term: control = 0.055±0.038, febuxostat = 0.278±0.039; µg/24 h; p>0.05 vs control). Febuxostat itself was successfully detected in samples from treated rats, including serum, liver and kidney (control rats serum, liver, kidney: undetectable; febuxostat-treated rats serum: 0.799±0.073, liver: 0.050±0.004, kidney: 0.038±0.012; µg/mg protein).

**Table 1:** HPLC measurements of uric acid and hypoxanthine in DOCA-salt rats after febuxostat administration.

(µg/mgprotein)	Short-term		Long-term	
	Control	Febuxostat	Control	Febuxostat
Serum uric acid	3.431±0.682	0.634±0.134*	0.788±0.210	0.074±0.020
Serumhypoxanthine	0.006±0.003	1.835±0.380*	0.000±0.000	0.604±0.144
Aorta uric acid	0.328±0.065	0.097±0.042	3.221±0.710	0.170±0.020
Aorta hypoxanthine	0.286±0.039	0.996±0.198	0.839±0.157	5.147±0.499*
Vena cava uricacid	1.883±0.702	0.255±0.112*	2.328±0.562	0.174±0.033
Vena cavahypoxanthine	0.309±0.131	0.614±0.159	0.271±0.043	1.932±0.415*
Liver uric acid	1.691±0.344	0.002±0.001*	9.715±1.692	0.046±0.005*
Liverhypoxanthine	0.066±0.028	1.147±0.109*	0.002±0.001	3.047±0.167*
Kidney uric acid	1.232±0.409	0.065±0.011*	10.850±2.75	0.221±0.031
Kidneyhypoxanthine	1.444±0.226	4.080±0.375*	0.784±0.287	9.041±0.736*

Data are expressed as means ± SEM for N = 6–8.

* represents a statistically significant difference (p<0.05) versus control rats.

### Effects of Febuxostat Administration on the Heart Weight, Kidney Weight and Media/lumen Ratio of the Aorta in DOCA-salt Rats

To observe potential BP-independent beneficial effects of febuxostat on renal, myocardial and vascular changes in DOCA-salt treated rats, we measured kidney and heart weight as well as aortic media/lumen ratio: all are typically increased in prolonged experimental mineralocorticoid hypertension. With the exception of a statistically significant reduction in aortic media/lumen ratio following long-term administration, febuxostat treatment did not modify these parameters, irrespective of the treatment duration (Table 2). Total body weight, used to normalize wet weights of organs, was not itself changed by febuxostat treatment (short-term: control BW = 359.57±24.94, febuxostat BW = 323.88±19.77; long-term: control BW = 377.38±15.24, febuxostat BW = 389.67±10.08; g; p>0.05).

**Table 2:** Effects of febuxostat administration on the heart weight, kidney weight and media/lumen ratio of the aorta in DOCA-salt rats.

	Short-term		Long-term	
	Control	Febuxostat	Control	Febuxostat
Heart weight (% body weight)	0.538±0.030	0.535±0.023	0.464±0.022	0.469±0.010
Kidney weight (% body weight)	1.000±0.107	0.992±0.062	0.778±0.026	0.814±0.081
Aorta media/lumen ratio	0.079±0.004	0.085±0.005	0.093±0.003	0.082±0.003*

Data are expressed as means ± SEM for N = 6–8.

* represents a statistically significant difference (p<0.05) compared to control rats.

### Febuxostat Incubation *in vitro* did not Modify Contractile Function of Aorta and Vena Cava

**Figure 3:**
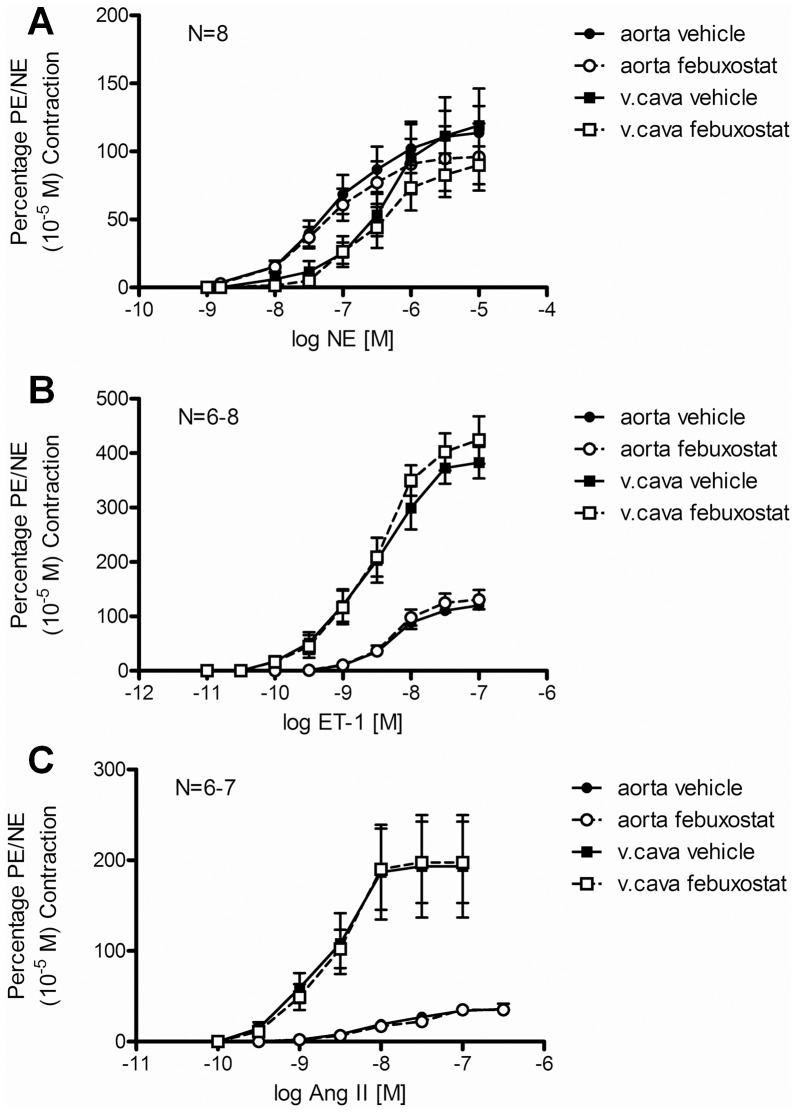
Cumulative concentration-response curves to norepinephrine (NE, A), endothelin-1 (ET-1, B) and angiotensin II (AngII, C) in aorta (circles) and vena cava (squares) after in vitro incubation with vehicle (full symbols) or 100 nM febuxostat (open symbols). Data are expressed as means ± SEM of initial contraction to phenylephrine (aorta) or NE (vena cava).

To evaluate whether xanthine oxidase inhibition by febuxostat has any direct effects on vascular contraction, we incubated aorta and vena cava rings from non-treated rats with febuxostat (100 nM) and measured contraction to norepinephrine (Fig. 3A), endothelin-1 (Fig. 3B) and angiotensin II (Fig. 3C). No significant change in sensitivity or maximal response was observed for any of these agonists (Table 3).

**Table 3:** Parameters of contractile force measurements represented in Figure 3: sensitivity (pD_2_) and maximal responses (E_max_) to norepinephrine (NE), endothelin-1 (ET-1) and angiotensin II (AngII) in aorta and vena cava after in vitro incubation with vehicle or 100 nM febuxostat.

	Aorta		Vena cava	
	Vehicle	Febuxostat	Vehicle	Febuxostat
NE pD_2_ (−logEC_50_, M)	7.19±0.05	7.26±0.04	6.41±0.04	6.53±0.05
NE E_max_ (% 10^−5^ M PE/NE contraction)	113.70±19.73	95.95±24.64	119.04±27.17	89.87±13.93
ET-1 pD_2_ (−logEC_50_, M)	8.20±0.08	8.20±0.09	8.56±0.05	8.51±0.04
ET-1 E_max_ (% 10^−5^ M PE/NE contraction)	120.33±7.18	130.70±17.66	382.68±28.95	424.20±43.64
AngII pD_2_ (−logEC_50_, M)	8.00±0.04	7.82±0.11	8.67±0.11	193.40±56.50
AngII E_max_ (% 10^−5^ M PE/NE contraction)	35.93±5.88	35.20±4.29	8.60±0.12	197.66±44.90

Data are expressed as means ± SEM for N = 6–8.

## Discussion

We have shown in this study that efficient inhibition of xanthine oxidase activity and decreases in tissue and circulating levels of uric acid by febuxostat (5 mg/kg/day) do not lower blood pressure in established hypertension or attenuate hypertension development in the rat DOCA-salt model. These results are in agreement with our previous findings on the lack of effects of allopurinol treatment (50 mg/kg/day) in the same model of hypertension [20], although arguably XO inhibition by febuxostat appears more profound than that achieved with allopurinol at the doses we used.

Previously published reports on the effects of XO inhibition by allopurinol on blood pressure have been contradictory, showing beneficial [15] or modest to no effects [14], [17], [18], [19], [20], [31]. Additionally, allopurinol has been shown to have non-specific effects on other enzymes, such as purine nucleoside phosphorylase and orotidine-5′-monophosphate decarboxylase, both also involved in nucleotide catabolism [22].

Febuxostat, a new XO inhibitor approved for administration in humans, has been recently demonstrated in clinical studies as superior in both safety and efficacy to allopurinol [23], [24], [25], [26], [32]. Moreover, Johnson *et al* showed that febuxostat decreased BP in hypertensive hyperuricemic rats fed a high fructose diet [28] as well as in the hyperuricemic hypertensive rats treated with the uricase inhibitor oxonic acid [29], [30]. However, no other studies have described febuxostat effects in a hypertension model not associated with hyperuricemia, such as the DOCA-salt model. This is the same hypertension model that we have previously used to test allopurinol [20] and the one in which the only blood pressure reduction was observed with allopurinol [15]. Furthermore, in contrast with the majority of studies mentioned above in which no or little validation of XO inhibition was performed, we have thoroughly assessed XO inhibition at the circulating and tissue level.

Febuxostat effectively inhibited XO activity systemically, as demonstrated by our measurements of XO metabolites. We presume that the decrease in uric acid levels observed in serum and at the tissue level was accompanied by a corresponding decrease in XO-generated ROS, however this would have to be validated in further studies. Since attenuation of overall ROS levels using either antioxidants or specific enzyme inhibitors decreased blood pressure in DOCA-salt [33], [34], [35] and other animal models of hypertension, our results would suggest that a specific decrease in XO-generated ROS alone is not sufficient for amelioration of blood pressure. Alternatively, febuxostat may inhibit xanthine dehydrogenase at a greater level than xanthine oxidase, thus generating a disparity between uric acid and ROS lowering effects.

DOCA-salt hypertensive rats have increased relative kidney and heart weight and increased arterial media/lumen ratio compared to sham uninephrectomized normotensive controls and these measurements are typically used as a gross evaluation of end-organ response to sustained hypertension. Febuxostat treatment did not decrease kidney or heart weight after either short or long-term administration. Aortic media/lumen ratio was decreased after long-term administration of febuxostat, raising the possibility of BP-independent effects of XO on the vasculature. However, in functional experiments, febuxostat incubation in vitro did not alter the contractile responses of aorta and vena cava to various contractile agonists, including ET-1 and angiotensin II, which are known for their ROS-mediated contractile effects [3]. The potential effects of febuxostat on hypertensive arterial remodeling may therefore be independent of effects on vascular contraction or of BP-effects.

We conclude that XO is not essential for either the development or the maintenance of hypertension in the rat DOCA-salt model. Our results therefore suggest that lowering uric acid levels is not sufficient to decrease blood pressure in the absence of hyperuricemia. This does not exclude other blood pressure-independent beneficial effects of febuxostat treatment in hypertension via a potential decrease of overall ROS levels, effects to be investigated in future studies.
